# Nonconsensual Sexual Experience Histories of Incarcerated Men: A Mixed Methods Approach

**DOI:** 10.1177/0306624X211065584

**Published:** 2021-12-19

**Authors:** Raymond M. McKie, Shulamit Sternin, Chelsea D. Kilimnik, Drake D. Levere, Terry P. Humphreys, Alyna Reesor, Elke D. Reissing

**Affiliations:** 1 University of Ottawa, School of Psychology, Ottawa, Ontario, Canada; 2 The University of Texas at Austin, Department of Psychology, Austin, Texas, USA; 3 The University of British Columbia, Department of Psychology, Vancouver, British Columbia, Canada; 4 Trent University, Department of Psychology, Peterborough, Ontario, Canada; 5 Department of National Defense, Canadian Armed Forces, Ottawa, Ontario, Canada

**Keywords:** nonconsensual sexual experiences, childhood sexual abuse, sexual assault, incarcerated men, inmates, prison, mental health

## Abstract

Nonconsensual sexual experiences (NSEs) may contribute to mental health concerns among incarcerated individuals, yet NSEs are understudied in this population. This study takes a novel approach in examining the prevalence of NSEs among incarcerated males by utilizing both quantitative and qualitative measures. The sample consisted of 189 men from three provincial maximum-security prisons in Ontario, Canada. Based on quantitative findings, 44.2% of the sample experienced NSEs before the age of 18, and 41.7% of the sample endorsed an experience that fit the legal definition of a NSEs as adults. Participants also responded to a qualitative open-ended question about their history of NSEs. Based on qualitative findings, a total of 23% of the men reported at least one incident of a NSE (e.g., child and adult). Based Findings highlight the high prevalence of NSEs among incarcerated men with quantitative responses demonstrating how the use of a behavioral questionnaire may, to some extent, correct for underreporting of NSEs. Qualitative responses illustrate the lived experience of incarcerated men and provide a deeper understanding of their NSEs. Responses also speak to the lack of resources and support available to these men. Findings underscore the need for proactive approaches in meeting mental health needs of incarcerated men in general and with regard to NSEs in particular. Results may inform the development of future correctional procedures (i.e., intake protocols that account for men with NSEs) and resources to support incarcerated men in navigating the psychological impact of non-consensual sexual experiences.

## Introduction

Incarcerated individuals experience high rates of mental health issues as compared to the general population (Brinded et al., 2001; Bronson & Berzofsky, 2017; Yi et al., 2017). For many incarcerated individuals these issues predate their confinement (Greenberg & Rosenheck, 2008; Mechanic & Rochefort, 1990) but the stress inherent in the carceral environment can exacerbate existing mental health problems as well as stimulate other issues (i.e., diminished sense of self-worth, post-traumatic stress reactions to the pains of imprisonment, emotional over-control, and psychological distancing; Haney, 2002; Kazemian & Walker, 2018). With some studies estimating that as many as half of all incarcerated individuals suffer from some form of mental health problem (James & Glaze, 2006) investigation into factors that influence psychological distress among incarcerated individuals has become an important topic of scholarly investigation (Bronson & Berzofsky, 2017; James & Glaze, 2006; Motiuk & Porporino, 1992; Skogstad et al., 2006; Yi et al., 2017).

One key factor that may contribute to mental health issues among incarcerated individuals is their history of non-consensual sexual experiences (NSEs; Ratkalkar & Atkin-Plunk, 2020; Wolff et al., 2007). The majority of literature addressing NSEs within incarcerated populations has focused on either history of childhood sexual abuse (CSA; e.g., Johnson et al., 2006; McGrath et al., 2011; Ogloff et al., 2012) or NSEs that occur during incarcerations (Neal & Clements, 2010; Ratkalkar & Atkin-Plunk, 2020; Wolff & Jing, 2009; Wolff et al., 2007). Both childhood and adult NSEs can hold serious mental health implications (e.g., alcohol abuse, suicide ideation and attempts, mood disorders, poor self-esteem) for incarcerated populations (Fondacaro et al., 1999; Neal & Clements, 2010; Ratkalkar & Atkin-Plunk, 2020; Struckman-Johnson & Struckman-Johnson, 2006; Wolff et al., 2007).

### Sexual Consent and NSEs

The absence of consent is a defining characteristic of NSEs (Beres, 2007; Mark & Vowels, 2020) and thus defining sexual consent has become an important area of research. An early and widely recognized definition is “the freely given verbal or nonverbal communication of feelings of willingness to engage in sexual activity” (Hickman & Muehlenhard, 1999, p. 259). Sexual consent has also been defined based on physical (Alexander, 1996; Ostler, 2003), psychological (Hurd, 1996), and emotional components (Hickman & Muehlenhard, 1999; Malm, 1996). More recently, sexual consent has evolved into the term affirmative consent (Shumlich & Fisher, 2018, 2020), which is sexual consent that is ongoing, continuous, and communicated by all parties to ensure that agreement to participate in a sexual interaction (i.e., “consent”) is freely given and voluntary (Burgin, 2019; Shumlich & Fisher, 2020). However, beyond this definition, affirmative consent is not clearly operationalized. For the purpose of being inclusive to a variety of forms of nonconsensual experiences, the current paper uses the term NSEs to describe any sexual interaction (or aspect of a sexual interaction) that occurs without freely given and ongoing sexual consent which may include, but is not limited to: rape, molestation, sexual assault, CSA, instances of coercion, incapacitation, or the use of force or threats to obtain sexual contact, abuse of power or authority, and instances where consent cannot be given (e.g., by a child with an adult or significantly older peer; Kilimnik & Meston, 2021).

### Labeling NSEs

Identifying NSEs with a sexualized violence label, such as “sexual abuse” or “rape,” is most commonly referred to in the literature as “acknowledgment,” (Cleere & Lynn, 2013) “labeling,” (Peterson & Muehlenhard, 2004) “defining,” and “identification” (Kilimnik et al., 2018). The psychological implication of identification is not clearly understood. Some research has found identification to be associated with decreased abuse related disability (Clements & Ogle, 2009), and those who identify with sexual violence labels are more likely to report the NSEs to the police, seek medical care, and utilize mental health services than those who do not identify with these labels (Wilson & Miller, 2016). Individuals who do not identify their NSEs with sexual violence labels have been found to be at greater risk for distress and psychopathology as compared to those who identify with these labels (Clements & Ogle, 2009; Littleton et al., 2009). However, several studies have also found that those who do identify with sexual violence labels report greater posttraumatic stress symptoms as compared to those who do not identify with these labels (Wilson & Scarpa, 2017). Lack of identification may be due to avoidance of a negative label (e.g., victim) but it may also be a result of individuals’ NSEs not matching their personal definitions for what characteristics of the experience or the perpetrator are required for the NSE to fit a given label (i.e., believing they should have fought back for the experience to constitute rape or sexual assault; Kilimnik & Meston, 2019; Peterson & Muehlenhard, 2004, 2011).

Many men with NSE histories do not identify their experiences with sexual violence labels (Artime et al., 2014; Holmes & Slap, 1998; Marsil & McNamara, 2016). In a pilot study of male college students, 5.2% of participants met the United States of America’s federal legal criteria for rape but only 1.4% identified their experience with the label of rape (Marsil & McNamara, 2016). In community samples of men, only 49% with CSA histories and 24% with rape histories used the labels of sexual abuse and rape, respectively to describe their NSEs (Artime et al., 2014). Of the limited research that has addressed identification among incarcerated men, 41% of incarcerated individuals who met the criteria for childhood NSEs did *not* identify their experience as sexual abuse and those men presented with higher rates of alcohol abuse and dependence compared to identifiers (Fondacaro et al., 1999).

Lack of identification among men can be understood within the context of masculinity norms and a socialization of men that emphasizes interpersonal autonomy (Purcell et al., 2004), to take the lead in sexual relationships (Saewyc et al., 2004), and to demonstrate high levels of sexual desire (Murray, 2018, 2019). NSEs contradict and undermine these norms (Javaid, 2017), which may threaten a man’s sense of power, control, and masculinity (Peterson et al., 2011; Saewyc et al., 2004). Further, rape myths (i.e., societal and community attitudes and false beliefs about rape that are widely and persistently held) and rigid definitions of masculinity may contribute to a lack of identification among men (Artime et al., 2014; Javaid, 2017) whose NSEs do not fit their personal definitions of given labels. A strong adherence to masculinity norms is especially prevalent in incarcerated men who have been found to report hyper-masculine ideologies and behaviors as compared to non-incarcerated men (Courtenay, 2000; Hua-Fu, 2005; Ricciardelli, 2015). In part, hypermasculinity, in this context, may be understood as a compensatory mechanism for a loss of identity within the prison hierarchies and power structure inherent in correctional facilities (Iwamoto et al., 2012; Michalski, 2017).

NSE identification has been associated with post-NSE adjustment trajectories in that identification of NSEs with sexualized violence labels may play a role in the integration of the experience into their cognitive representation of their sexual selves and ultimately impact their wellbeing (Kilimnik et al., 2018). Men who do not identify their NSEs may face challenges resolving their experience with the ideals of masculinity, which can manifest as intimacy issues, emotional discomfort, alienation, and anger (Kia-Keating et al., 2005). Due to the language used in recruitment efforts and measurement tools (e.g., “sexual abuse,” “rape”), individuals who identify their NSEs with common sexualized violence labels have historically been the primary subjects of research (Kilimnik & Meston, 2019; Koss, 1985). As such, using behavioral measures and broader recruitment language is critical to be inclusive of those who both do and do not identify their NSEs with sexual violence labels (Kilimnik et al., 2018).

### NSEs and Incarceration

The social determinants of health among incarcerated men such as poverty, limited opportunities for formal education, difficulties with housing, unemployment, and racial and ethnic disparities (Stewart et al., 2018) that marginalized populations and increase the likelihood of criminal activities (Woodall et al., 2014) are factors often found to be comorbid with poor mental health among incarcerated individuals (Trotter et al., 2018). Further, there is an overlap between the factors associated with a child’s increased risk for later criminal behavior and those associated with an increase of childhood NSEs (Chung et al., 2002; Ou & Reynolds, 2010; Sitnick et al., 2017; Sourander et al., 2006). In a population-based study, 27.3% of child maltreatment was attributable to economic disadvantage (i.e., poverty and parental unemployment; Doidge et al., 2017), parental substance use, and social instability (Doidge et al., 2017; MacMillan et al., 2013). While many factors have been identified with an increased risk for adulthood NSEs, such as alcohol use, illicit drug use, and psychological distress (Elliott et al., 2004; Rich et al., 2004), one well documented predictor of adulthood NSEs is childhood NSEs (Desai et al., 2002; Filipas & Ullman, 2006; Han et al., 2013; Messman-Moore et al., 2000; Walker et al., 2019). This phenomenon is often referred to as sexual revictimization (Walker et al., 2019). In a national sample of men, those who experienced adulthood NSEs were five times more likely to have a history of childhood NSEs than matched counterparts (Elliott et al., 2004). Men with repeated experiences of NSEs have also been found to report greater levels of psychological distress than those with a history of childhood or adulthood NSEs alone and as compared to those with no NSEs (Elliott et al., 2004).

The rate of childhood NSEs is higher within incarcerated populations as compared to the general population (Fondacaro et al., 1999; Johnson et al., 2006; Ogloff et al., 2012; Weeks & Widom, 1998). In longitudinal studies that followed individuals with CSA histories and matched controls, those with CSA histories were found to be five times more likely to be charged with a criminal offense and significantly more likely to be a victim of sexual offenses later in life (Ogloff et al., 2012). Early examination of police reports found that 11% of incarcerated men had reported childhood NSEs (Dutton & Hart, 1992). In a study that assessed childhood NSEs among incarcerated men through a battery of “yes” or “no” questions regarding various sexual activities, that did not require participants to identify their NSEs with any sexual violence labels, it was found that 40% of men experienced sexual activity with an individual at least 5 years older, when under the age of 16 (Fondacaro et al., 1999). In a study using the National Health and Social Life Survey (Laumann et al., 1994) that assesses NSEs without using sexualized violence labels, 59% of incarcerated men in a Southeaster Texas county jail reported experiencing some form of sexual abuse before puberty (Johnson et al., 2006). Lack of consistent operational definitions and measurement of childhood NSEs (Gorey & Leslie, 1997; Johnson et al., 2006; Ogloff et al., 2012) may account for some of the variation in prevalence rates. Despite these variations, childhood NSEs are believed to be a key risk factor in criminal behavior (Ardino, 2012; Wolff et al., 2017). One qualitative investigation found that instances of childhood NSEs and a lack of support and/or treatment was common in trajectories leading to incarceration (Honorato et al., 2016). One explanation for this association is the multitude of shared risk factors associated with both childhood NSEs and risk for later criminal behavior (i.e., poverty, lower parental education, family instability; Chung et al., 2002; Ou & Reynolds, 2010; Sitnick et al., 2017; Sourander et al., 2006). Another interpretation is that those with child NSEs often have difficulties developing strong interpersonal relationships and social ties (Pears & Capaldi, 2001) and are at a higher risk for psychopathology (e.g., PTSD, suicide, depression, anxiety, low self-esteem, substance abuse, eating disorders, and personality disorders; Hill et al., 2000), which may contribute to offending propensity.

As compared to men in the general population, a higher rate of adulthood NSEs has also been reported among incarcerated men with most of the literature addressing NSEs that occur during incarceration (Hensley et al., 2003; Struckman-Johnson & Struckman-Johnson, 2000, 2006; Wolff et al., 2006, 2007). Like childhood NSE research, variability in methodological approaches have resulted in a wide range of prevalence rates. For example, studies using face-to-face interviews tend to report lower rates of NSEs during incarceration (e.g., Lockwood, 1980; Nacci & Kane, 1983) in comparison to anonymous surveys (e.g., Struckman-Johnson et al., 1996; Wooden & Parker, 1982). Prevalence rates of NSEs during incarceration have been found to range from 3% (of 60,000 participants; Davies, 1982) to 28% (of 89 participants; Lockwood, 1980). Others have reported rates of 11% (of 330 participants; Nacci & Kane, 1983) while a more recent study has reported a rate of 14% (of 173 male participants; Hensley et al., 2003). Incarcerated individualss who have NSEs during incarceration may experience physical injury, trauma responses, anxiety, and hopelessness. Further, failure to disclose the incident can lead to a lack of detection and treatment for sexually transmitted infections, depression, and suicide (Dumond & Dumond, 2002). To the best of our knowledge, there is no study to date that has addressed NSEs across the lifespan of incarcerated men, that occurred both prior to and during incarceration.

## Purpose

Elevated prevalence rates of childhood and adulthood NSEs among incarcerated men coupled with the known negative psychological consequences of NSEs and a high rate of mental health issues among incarcerated individuals highlight the need to better understand the history of NSEs of incarcerated men. While previous research has addressed childhood NSE histories and NSEs during incarceration, there has been limited qualitative investigation into both childhood and adulthood NSE histories among incarcerated men. A mixed-methods approach to examining NSE histories provides a holistic picture of the challenges faced by this population and can be used to better inform resources as well as clinical and research initiatives. With the responsibility to rehabilitate incarcerated individuals placed on the carceral system, addressing NSEs is an important area of focus for correctional educational programing, a known effective form of intervention for mental health outcomes among incarcerated individualss (Byrne, 2020; van der Linden, 2015).

The first objective of the study was to qualitatively explore, through an open-ended question, the lived experience of incarcerated men’s sexual boundary violations. The second objective was to examine self-reported NSE histories within male incarcerated populations using a question that addressed CSA along with a validated scale (Sexual Experience Survey- Short Form Victimization; SES-SFV; Koss, 2006) addressing adult NSEs.

## Methods

### Participants

A total of 189 participants were recruited from three maximum-security correctional centers in Ontario, Canada: Maplehurst Correctional Complex (MHCC) (*n* = 60, 30.2%), Ottawa-Carleton Detention Center (OCDC) (*n* = 45, 22.6%), and Central East Correctional Center (CECC) (*n* = 84, 42.2%). While all men completed the quantitative scales, three men did not complete all qualitative questions. All three facilities are provincially run and house incarcerated individuals who are either convicted or on remand (awaiting trial). Canadian provincial and territorial run prisons are generally a place for incarcerated individuals who have sentences of 2 years or less however, due to prison overcrowding and deferral of court proceedings many incarcerated individuals detained in provincial or territorial prisons may be serving a longer sentence. The men ranged in age from 19 to 74 (*M* = 35.13). Most participants were born in Canada (*n* = 149, 74.9%) and identified as White or from European descent (*n* = 141, 71%). A further 15 participants (8%) identified as African, Caribbean, or Black and 13 (6.5%) identified as Indigenous. A total of 107 participants (53.8%) reported having a high school degree or less, 41 participants reported completion of some college/university, and 26 participants reported completion of a post-secondary degree or diploma. All participants self-identified as male. The majority of the participants reported being heterosexual (*n* = 168, 94.4%), with 11 (5.5%) participants reporting being gay, bisexual, or questioning (see Table 1 for further demographic information).

**Table 1.** Self-Reported Demographic Information.

	Frequency	Percent
Participants		
Maplehurst correctional complex	45	23.8
Ottawa-Carleton Detention Center	60	31.7
Central East Correctional Center	84	44.4
Ethnicity		
Aboriginal	13	8.1
Asian (South, South-East)	7	4.4
Black (Africa, Canadian, Caribbean)	14	8.8
Indian	1	0.6
Middle Eastern	1	0.6
White (Great Britain/Irish, Eastern European, Italian, other European, other)	124	77.5
No response	29	
Education		
Not sure	2	1.1
Less than high school	43	24.0
High school	64	35.8
Some college/university	41	22.9
College/university	26	14.5
Graduate school	3	1.7
No response	10	
Sexual orientation		
Straight/heterosexual	168	93.9
Gay	2	1.1
Bisexual	8	4.5
No sure/questioning	1	0.6
No response	10	
Religion		
Catholic	71	40.3
Protestant	23	13.1
Eastern orthodox	4	2.3
Other Christian	21	11.9
Muslim	9	5.1
Hindu	1	0.6
Sikh	1	0.6
Buddhist	2	1.1
Agnostic	5	2.8
None	27	15.3
Other	12	6.8
No response	13	

*Note.* Percentages calculated based on total number of respondents to the given question.

### Procedure

Research Ethics Board approval was granted by the Ontario Ministry of Community and Correctional Services/Correctional Services Canada.

#### Participant recruitment

With the aid of correctional social workers and psychologists to gain entry into the prisons, research assistants visited populated cells and pods (i.e., 4–6 cells consisting of incarcerated individuals with similar offenses) with sign-up sheets to invite incarcerated individuals to participate in a study on sexuality and relationships. The Ministry of Community and Correctional Services does not allow compensation of incarcerated individuals for research studies; thus, no compensation was provided to participants (Hanson et al., 2012; Matheson et al., 2012). It should be noted that participation in the study did replace program attendance or work for incarcerated individuals and provided an opportunity to spend time away from other incarcerated individuals during data collection, which many participants have noted as reasons for participating in other research (Hanson et al., 2012). Research assistants reviewed the consent form with interested participants in a private interview room. Those that consented then completed the survey independently. Research assistants provided additional information for participants that had questions pertaining to items or wording concerns. Only a few participants required a research assistant to verbally administer the questionnaire due to difficulties reading.

Data collection began at the Central East Correctional Center (CECC) and as the project progressed two other facilities were added: Maplehurst Correctional Complex (MHCC), and Ottawa-Carleton Detention Center (OCDC). The expansion of the study to include these two new facilities was the result of two authors moving locations and the initial success of the project at Central East. During REB renewals, the survey package given to the incarcerated individuals was updated to include a question on child abuse and an additional qualitative question pertaining to the desire/need for institutional programing regarding sexuality issues. At this time, participant recruitment had stopped at CECC (*n* = 84). Thus, this additional data came from participants located at OCDC (*n* = 45) and MHCC (*n* = 60).

### Measures

Data were drawn from a larger data set assessing attitudes, beliefs and values pertaining to sexual consent and boundary setting among men in Ontario prisons. To assess childhood abuse history, participants responded to the question “Have you ever experienced any of the following forms of abuse in your childhood?” and were able to select all options that applied from a list: sexual abuse, physical abuse, emotional abuse/neglect. Participants also indicated, if possible, the age at which the abuse occurred: before the age of 14 (but not after), after the age of 14 (but before 18), both before and after the age of 14 (but before 18), No, Unsure.

In addition, participants also completed the behavior-specific Sexual Experiences Survey Short-Form Victimization (SES-SFV; Koss et al., 2007). The SES-SFV is a 10-item tool used to estimate the frequency and type of unwanted sex act and/or the rate at which given tactics to compel nonconsensual sex have occurred (Koss, 2006; Koss et al., 2007). The SES-SFV is one of the most widely used measures of NSEs and exhibits good reliability (Anderson et al., 2018), as well as adequate convergent validity with self-reported sexual coercion by a romantic partner and childhood sexual abuse among male survivors and the Sexual Coercion subscale of the Revised Conflict Tactics Scales (Anderson et al., 2018; Johnson et al., 2017). Qualitative data was collected from participants responded to the open-ended question: “Have you ever been in a sexual situation where you felt your own sexual boundaries (sexual limits that you have set for yourself) have been breached or broken? If so, please describe the situation and how you dealt with it.”

### Data Analysis

#### Quantitative analysis

Quantitative data collected from the questions addressing CSA and the SES-SFV were analyzed using R Studio software, Version 1.3 (RStudio Team, 2020). The frequency of responses to each question addressing CSA were calculated. The SES-SFV was scored according to the guidelines outlined by Koss et al. (2007), which uses the five perpetrator tactics of each of the five behaviorally-specific items to classify individuals’ NSEs into nonconsensual sexual contact, attempted coercion, coercion, attempted rape, and rape. Categories are not mutually exclusive, such that individuals may have experienced more than one type of NSE.

#### Qualitative analysis

The qualitative responses were analyzed following Braun and Clarke’s (2006) six phase thematic analysis guidelines by two researchers with relevant experience in forensic psychology and sexuality. Thematic analysis is a method for identifying, analyzing, and reporting patterns (themes) within data that can provide a rich and detailed, yet complex account of the data.

1. The two data coders initially familiarized themselves with the data, reading and re-reading participant responses.
2. Initial codes which encompassed features of the data in a systematic fashion across the entire dataset were then generated.
3. All data relevant to the central research question of the study were coded, these codes were then collected into general themes. Coding was completed using NVivo qualitative analysis software.
4. The general themes were then checked in relation to both the coded extracts and the entire dataset that allowed for a thematic “map” of the analysis.
5. Defining and naming the themes was achieved through review and discussion between the two analysts. Themes were reviewed based on data saturation and those that did not hold a sufficient number of relevant quotes were removed from the thematic map, others were merged due to coding overlap (Braun & Clarke, 2021). The thematic map was understood to comprehensively encompass all themes once saturation was reached and all quotes had been coded indicating that further analysis was unnecessary (Saunders et al., 2017). To determine the inter-rater reliability of the coding of extracts within each defined theme a Cohen’s Kappa value was calculated (κ = .78), indicating a substantial level of agreement between raters (McHugh, 2012).
6. Vivid and compelling extract examples relating back to the research question and literature were selected in the final stage of analysis (Braun & Clarke, 2006). Presented quotes have been edited to remove spelling and grammatical mistakes while maintaining the integrity and explicit meaning of the quotes.

## Results

### Quantitative Results

Sixty-eight participants responded to the question addressing CSA, 44.2% reported having experienced sexual abuse before the age of 18. Seventy-nine participants responded to the question addressing child physical abuse with 58.3% experiencing physical abuse before the age of 18. Seventy-four participants responded to the question addressing emotional abuse with 58.6% experiencing emotional abuse before the age of 18 (See Table 2). Of the 189 respondents who completed the SES-SFV 83 (49.4%) reported NSEs (Table 3). Of the 83 participants those who reported NSEs, 45 (26.7%) endorsed items that characterized rape, 35 (20.8%) attempted rape, 32 (19.1%) coercion, and 25 (14.9%) attempted coercion (categories are not mutually exclusive).

**Table 2.** Self-Reported Child Sexual Abuse.

	Frequency	Percent
Type of abuse (not mutually exclusive)		
Sexual (total number of responses to this question)	68	
No	38	55.9
Yes—before the age of 14	18	26.5
Yes—after the age of 14 (but before 18)	1	1.5
Yes —both before and after the age of 14 (but before 18)	12	17.7
Physical (total number of responses to this question)		
No	79	
Yes—before the age of 14	31	39.2
Yes—after the age of 14 (but before 18)	18	22.8
Yes—both before and after the age of 14 (but before 18)	6	7.6
Unsure	22	27.9
Emotional (total number of responses to this question)	2	2.5
No	74	
Yes—before the age of 14	29	39.2
Yes—after the age of 14 (but before 18)	10	12.7
Yes—both before and after the age of 14 (but before 18)	4	5.4
Unsure	30	40.5
	1	1.4

**Table 3.** Sexual Experiences Survey—Short Form Victimization (SES-SFV) Results.

	Frequency	Percent
Any report on the SES-SFV		
Yes	83	49.4
No	85	50.6
No response	31	
Not mutually exclusive categories		
Rape	45	54.2
Attempted rape	35	42.2
Coercion	32	38.6
Attempted coercion	25	30.1
Mutually exclusive categories		
Sexual contact	23	27.7
Attempted coercion	2	2.4
Coercion	7	8.4
Attempted rape	6	7.2
Rape	45	54.2

*Note.* Percentages calculated based on total number of respondents that had any report on the SES-SFV.

### Qualitative Results

Data analysis was concluded with a model of three major categories: *Childhood NSE (*divided into two themes, *Adult Perpetrator, Child Perpetrator), Adulthood NSE (*divided into three themes, *Adult Sexual Assault, Harassment, and Unwanted Sexual Acts)*, and *No NSE.* A total of 49 participants (25.9%) described a situation in which their sexual boundaries had been breached or broken, 78 (41.3%) responded with “No” or other variants that indicated they had not experienced a sexual boundary violation (e.g., “Never happened as I was always in control”), and 59 (31.2%) left the question blank.

#### Childhood NSEs

##### Adult perpetrator

Incarcerated men reported instances of childhood NSEs and specified that the perpetrator of the NSEs was an adult and in many cases a person familiar to the incarcerated individual. Perpetrator familiarity was described by one incarcerated individual as: “Once when I was sleeping my uncle came and slept beside me, I woke up with him touching me, but I never once told anyone because he was drunk as hell” (Participant 4, Age 38). Another incarcerated individual wrote:

When I was 12 years old, I woke up because I felt someone touching my penis, it was my uncle’s friend. He told me to not say anything or yell and continued masturbating and sucking on my penis until I came (Participant 26, Age 47).

There were a few incarcerated individual who addressed the fact that their NSE is still an issue for them today. For example, one described: “When I was 9 years old my stepdad did molest me as a child. I grew up dating people and was very angry in life. And still feel I have ongoing problems because of this incident” (Participant 142, Age 30). A father figure perpetrator was also described by another incarcerated individual who wrote:

Yes, when I was a young kid, my father would drug me and my brother and touch us and my mom couldn’t stop it because my father would beat her and us if we said anything. My mom got fed up and ran away with me and my brother and I haven’t seen him since (Participant 148, Age 36).

Other incarcerated individuals described experiences where the perpetrators were parents of their friends, for example one incarcerated individual said “Yes, when I was young and at a girl’s house her mom convinced us to touch each other sexually” (Participant 71, Age 24). Another recollected that:

I was on my way home and I got a ride by my friend’s dad and he wanted it to give me a blowjob and I told him no and he did not take no and put his hands on me, so I jumped out of his car and went to the cops. The cops did fuck all about that shit that my friend’s dad did to me (Participant 35, Age 41).

As the previous quote illustrates, there were many responses that addressed the lack of support received after their NSEs and their inability to manage the consequences of the experience. One respondent wrote: “My stepmother molested me for approximately 18 months, I tried to report it to the police years later. At the time I used and abused myself and drugs” (Participant 17, Age 35). Another stated:

Never knew how to deal with it, plus I don’t trust anyone enough. But yes, when I was 14 and 16, I was forced into sex by a man that involved drinking and drugs and being homeless. I was scared and felt I had no choice (Participant 47, Age 31).

One incarcerated individual expressed the lack of support they received and their challenges in managing their NSE saying:

Yes, when I was a kid, 12, I was abused by a friend who was 18, with a plastic toy sword. I didn’t know how to process/ deal with that, so I blocked it out, mentally, until last year. I’m still struggling with how to deal with this as I haven’t received any counseling (Participant 187, Age 23).

Some incarcerated individuals described childhood NSEs but did not specify who the perpetrator was. For example, one incarcerated individual wrote: “I’ve never had this issue as an adult. But I was raped as a young boy. It happened between the ages of 4 and 7” (Participant 3, Age 38). Another recounted a kidnapping story:

I was kidnaped at age 6 and sexually assaulted. At age 16 I was forcibly confined and had my eyes stabbed with the butt end of a knife. The attacker then performed anal sex on me and threatened to cut off my penis because I couldn’t get an erection (Participant 87, Age 42).

##### Child perpetrator

Incarcerated individuals also reported childhood NSEs with child perpetrators and, similar to responses regarding adult perpetrated NSEs, child perpetrators were often people familiar to the men. For example, one incarcerated individual said: “Yes, I was sexually abused by an older female cousin when I was 8 years old, and it went on for almost a year. There was oral sex, touching and she acted like she was my teacher” (Participant 91, Age 29).

Some incarcerated individuals spoke to a peer pressure component of their childhood NSEs. For example, one incarcerated individual said:

An older boy convinced me and a younger girl to show each other our private parts. I was about 10 at the time, I never really talked about it with anyone or dealt with it properly. Part of the reason is I feel guilty because I enjoyed it even though I knew it was wrong (Participant 71, Age 24).

Another stated: “When I was 16, I was given anal sex in front of others. I just went with it because I wanted to fit in” (Participant 48, Age 36). Similar to participants who described adult-perpetrated childhood NSEs, some responses illustrating child-perpetrated NSEs spoke to a lack of support following their experience, for example one incarcerated individual wrote: “Yes I was grabbed by a fellow student while in grade school in the groin region. He called the grabbing of my crotch “strang”[sic] and acted like it was a joke. I told school administrators, but nothing was done” (Participant 80, Age 36). Another incarcerated individual revealed that this was the first time they had disclosed their experience saying: “Older stepbrother sexually abused me often as a child. Never told anyone. Still haven’t. This is the first time. No one ever asked” (Participant 49, Age 43). While respondents were not asked about the sequelae of their NSEs one incarcerated individual described the long-term consequences saying:

[The experience has] affected me up through most of my life. This affected three marriages which all ended sadly. I was to blame for this as I now know that I was still a small boy when it came to sex. I was a man with the needs of a man, but the horror of sex sickened me (Participant 115, Age 49).

#### Adulthood NSEs

##### Adult sexual assault

Some incarcerated individuals who spoke to adult NSEs described sexual assault, such as unwanted touching by another male. For example, one incarcerated individual said:

Yes, I have been, I was put in a situation where I was “touched” by another male, the incident only took place once and I have disliked the feeling ever since. I dealt with it by keeping it to myself and just moved forward in life (Participant 39, Age 33).

Another described an experience that occurred in a public place saying: “I was at a men’s bathhouse in Toronto and an Asian man tried grabbing my privates, so I pushed him away and said he had no right to touch me” (Participant 128, Age 50).

##### Unwanted sexual acts

Multiple incarcerated individuals described adulthood NSEs that involved a sexual interaction where anal penetration occurred or was attempted. For these incarcerated individuals this type of sexual act clearly crossed their sexual boundaries. One incarcerated individual said: “Yes, I had a girlfriend who was sometimes interested in sticking finger(s) in my anus. The way she explained herself to me was that she thinks that all men like it and there is no shame in that. I simply don’t feel comfortable letting even a female who appears to be my girlfriend touch my “anus” ” (Participant 43, Age 28,). Other incarcerated individuals echoed this experience with one saying, “My girlfriend tried to put a finger in my ass while having oral sex. I wasn’t comfortable” (Participant 131, Age 51). Another incarcerated individual described their experience by saying: “[I] just went with the flow of things when my girlfriend decided to put something in my ass. I began to enjoy it” (Participant 95, Age 32).

Other incarcerated individuals spoke to experiences where their sexual partners asked for sexual acts that they did not feel comfortable performing for example, one incarcerated individual stated:

Yes, my girlfriend likes to be choked which I am OK with, she lets me know when to stop but she asks me to hit her and that is just a bit too much for me. I have done the slap a few times but not hard enough for her and too much for me to get off (Participant 143, Age 30).

Another described a similar experience but indicated that this was not necessarily a crossing of their sexual boundaries:

Not that I could say my boundaries were broken but I have been asked to do things to a person I didn’t feel completely comfortable with. . .. For example, one girl wanted to have me choke her while having sex and I didn’t feel comfortable (Participant 73, Age 24).

Another incarcerated individual spoke to an unwanted sexual experience that occurred within the context of a same-sex sexual interaction:

At times while doing drugs and hooking up with men, there have been times when the other was too dominant or vocal with desires. That would bring forth in me feelings of self-disgust or shame. I was more comfortable with less talk about the actions occurring (Participant 156, Age 52).

##### Harassment

Other responses that spoke to adult NSEs addressed incidents of harassment. For example, one incarcerated individual stated that: “. . .two homosexual males made lewd comments about me while on a bus. Had to get off the bus” (Participant 80, Age 36). Another incarcerated individual recalled a similar experience of being labeled as gay:

Being called gay and having other males try to rub up against me, particularly while in jail. I dealt with it by just brushing it off and walking away. They have since stopped doing it to me after I told them several times that I am not gay. I have no problem with others being gay, but I don’t like being labeled as such. (I have been bullied in this fashion since junior high) (Participant 117, Age 24).

#### No NSE

A total of 41% of participants reported that they had not had NSEs. Many of their responses spoke of being in control of their sexual boundaries. For example, one participant stated, “My sex life is under my control for the most part and partners have an understanding of my sexual desires and boundaries” (Participant 46, Age 21). Knowing one’s sexual boundaries was echoed by another incarcerated individual who wrote: “There were times in my life when I had many different sexual partners and boundaries were something put in place. They were always respected both ways” (Participant 145, Age 31).

One incarcerated individual spoke to knowing their own limits: “Never. I know my limits, but I’ve never been in a situation where I felt the need to say stop or what not” (Participant 84, Age 37).

Another incarcerated individual addressed how they go about navigating a situation in which they felt their boundaries being crossed:

When I have encountered a situation (sexually) I was uncomfortable with, I openly discussed my feelings and limitations with my partner, and we came to an equal understanding of the most appropriate course of action. Receiving input from the person you are having a sexual relationship with is important as it gives them greater insight into your feelings as well as their own (Participant 86, Age 28).

## Discussion

The aim of the current study was to explore the NSE histories of incarcerated men through both qualitative and quantitative measures to gain a holistic understanding of their experiences. The body of literature addressing NSEs in this population has been limited by its focus on either childhood NSEs, or adulthood NSEs that occur during incarceration. Further, the majority of studies have addressed NSEs among men who identify their experience with sexualized violence labels. The current study takes a novel approach in addressing NSE histories among incarcerated men by using both qualitative and quantitative measures of NSEs to provide unique insights into the experiences of incarcerated men. Through qualitative investigation, 23% reported NSEs, while quantitative investigation revealed that 44.2% of the sample had experienced childhood NSEs before the age of 18, and 49.4% had experienced adult NSEs. These results indicate high rates of both child and adult NSEs among incarcerated men as well as highlight how qualitative investigation of NSEs may result in underreporting of experiences in this population.

Qualitative responses were organized into three categories: *Childhood NSEs* (divided into two themes, *Adult Perpetrator, Child Perpetrator), Adulthood NSEs* (divided into three themes, *Adult Sexual Assault, Harassment, and Unwanted Sexual Acts*), and *No NSEs.* Within our sample, 23% of participants qualitatively reported NSEs. This finding stands out from previous research where rates for adulthood heterosexual men’s NSEs ranged between 2-9% (Lockwood, 1980; Nacci & Kane, 1983; Sorenson & Siegel, 1992; Yuan et al., 2006) and 5% to 11% for childhood NSEs (May-Chahal & Cawson, 2005; Ross et al., 2005; Siegel et al., 1987) when qualitatively examining NSEs among men in the general population. Of the limited research that has assessed both childhood and adulthood NSEs among men in the general population, prevalence rates have been found to be around 8% (Coxell et al., 1999). Both childhood (i.e., Johnson et al., 2006) and adulthood (i.e., Peterson et al., 2011) NSEs are known to be higher among incarcerated populations as compared to the general population, and thus a rate of 26% of the current sample reporting some instance in which their sexual boundaries had been breached or broken fits within the extant literature. It must also be taken into consideration that qualitative responses spoke to both childhood and adulthood NSEs, with adulthood NSEs not being limited to experiences that occurred during incarceration. Previous research was designed to investigate either childhood or adulthood NSEs; and adulthood NSEs were typically limited to those occurring during incarceration.

Quantitative responses to the question addressing CSA revealed that 44.2% of the sample had experienced CSA before the age of 18, a rate consistent with that found in the literature on incarcerated men (Fondacaro et al., 1999; Johnson et al., 2006). Qualitative responses that described childhood NSEs illustrated the nature of the CSA, with most incarcerated individuals reporting experiences where the perpetrators, be it a child or adult, were familiar to the incarcerated individual (e.g., father, cousin). The level of familiarity with the perpetrator can have a significant influence on the effect of NSEs, with mental health outcomes of childhood NSEs often being more severe for those with perpetrators that are close to and subsequently trusted by the child compared to those with perpetrators that are less trusted (Davies et al., 2013; Edwards et al., 2012; Finkelhor & Browne, 1988; Ullman, 2007). Given that childhood NSEs are a key risk factor for future NSEs (Desai et al., 2002; Filipas & Ullman, 2006; Han et al., 2013; Messman-Moore et al., 2000; Walker et al., 2019), and that incarcerated men are at a high risk of NSEs during incarceration (Hensley et al., 2003, 2005; Struckman-Johnson & Struckman-Johnson, 2000, 2006; Wolff et al., 2006, 2007) it is important that childhood NSEs be taken into consideration upon entry into the carceral system and addressed by targeted interventions in order to help incarcerated individuals avoid adult revictimization.

Using the SES-SFV, adulthood NSEs were reported by 49.4% of the men in the present sample–a rate more than twice that of prior studies for adulthood NSEs among incarcerated men (Hensley et al., 2003, 2005; Struckman-Johnson & Struckman-Johnson, 2006; Tewksbury, 1989; Wolff et al., 2006). We suggest two possible explanations for these results. The first is the use of the SES-SFV, a tool designed to measure NSEs that meet most legal definitions of sexualized violence with behavior-specific items but does not require respondents to identify their experiences with sexual violence labels. To the best of our knowledge the current study is the first to use the SES-SFV with a male incarcerated population and thus may be reporting on NSEs experiences that previous measures did not capture. A second explanation for the high rate of adulthood NSEs in our sample can be attributed to not limiting assessment of NSEs to experiences that occurred during incarceration but instead included all adulthood NSEs that may have occurred prior to and/or during incarceration.

The discrepancy between qualitative reports of NSEs and quantitative reports of NSEs in our sample demonstrates that the use of an objective behavioral measure such as the SES-SFV as compared to a subjective measure, such as the qualitative question used in this study, may be a more accurate tool for identifying those who have had NSEs within an incarcerated population. incarcerated individuals may be underreporting NSEs due to shame, fear of repercussions, internal conflict, or alternative reasons. Despite this underreporting 23% of participants did qualitatively report NSEs which is a rate significantly greater than qualitative reports of heterosexual men in the general population that have found adulthood NSEs to range between 2% to 9% (Lockwood, 1980; Nacci & Kane, 1983; Sorenson & Siegel, 1992; Yuan et al., 2006) and 5% to 11% for childhood NSEs (May-Chahal & Cawson, 2005; Ross et al., 2005; Siegel et al., 1987).

Men’s NSEs contradict hegemonic definitions of male sexuality that require men to be, dominant and in control to initiate and pursue sex (Muehlenhard et al., 2016; Ricciardelli, 2015; Weiss, 2010), and to demonstrate high levels of sexual desire (Murray, 2019). These expectations on men and the known hyper-endorsement of masculine ideology among male incarcerated individuals (Courtenay, 2000; Hua-Fu, 2005; Ricciardelli, 2015), may contribute to qualitative underreporting of NSEs and may *partially* explain the 77% of participants who chose to not respond to the qualitative question. It is possible that within this non-reporting group there may be individuals who are not reporting their NSEs.

The majority of incarcerated individuals’ qualitative responses described instances of sexual harassment, inappropriate touching, nonconsensual acts that occurred during a sexual interaction, as well as other experiences in which incarcerated individuals felt their sexual boundaries had been crossed (e.g., being asked to choke or hit a partner). Many responses that described adulthood NSEs spoke to instances of unwanted sexual acts that involved anal stimulation. Strong heterosexual sexual-script endorsement and hyper-masculine ideals and behaviors among incarcerated men (Courtenay, 2000; Hua-Fu, 2005) may result in the discomfort with any association of stereotypically gay behaviors and male same-sex sexual activity. As one inamate stated, “I have no problem with others being gay, but I don’t like being labeled as such.” This rejection of male same-sex sexual interactions may be rooted in the hyper endorsement of heterosexual masculinity (Murray, 2018) and the strong emphasis in correctional institution culture on the maintenance of masculinity (Hensley et al., 2003).

The known impact of both childhood and adulthood NSEs on mental health (particularity if left unaddressed), can be compounded by the known lack of identification and disclosure of NSEs among men (e.g., Walfield, 2021) and the incarcerated male reluctance to seek help for mental health issues (e.g., Skogstad et al., 2006). Despite this, 23% of participants chose to disclose their NSEs in writing to the open-ended questions which speaks to the willingness of participants to share their experiences and in some cases to seek help under conditions that protect anonymity. As one participant stated, “I didn’t know how to process/deal with that, so I blocked it out, mentally, until last year. I’m still struggling with how to deal with this as I haven’t received any counseling” (Participant 187, Age 23). Some incarcerated individuals also reported that they had never disclosed their NSEs or that their experience had never been addressed by a medical or mental health professional.

Studies of both childhood and adulthood NSE survivors have found that delayed disclosure can have many negative effects on psychological symptoms (Ullman, 2007; Ullman et al., 2007) as disclosure of NSEs may serve to assist in the recovery process (Tener & Murphy, 2015). It has been suggested that a lack of support in coping with and managing NSEs may be a contributing factor in the maladjustment of incarcerated men (Connolly & Woollons, 2008; Gewirtz-Meydan & Opuda, 2022; Hornor, 2010). One incarcerated individual stated that this was the first time they had disclosed their experience saying “Never told anyone. Still haven’t. This is the first time. No one ever asked” (Participant 49, Age 43). These responses illustrate the lack of systemic support in the carceral system in addressing NSE histories in prison incarcerated individuals. For example, when entering the prison system in Canada there is no protocol for assessing history of NSEs and this lack of inquiry and subsequent support and intervention may have detrimental repercussions on incarcerated individuals that affect mental health. The social determinants of health among incarcerated men (i.e., limited opportunities for formal education, difficulties with housing, unemployment, and racial and ethnic disparities; Stewart et al., 2018) that marginalize this population and increase the likelihood of criminal activities (Woodall et al., 2014). Marginalized individuals or those with fewer socioeconomic opportunities may be at a higher risk of NSEs and this may contribute to the higher rate of incarceration and mental health issues among these individuals (Trotter et al., 2018).

Lack of NSEs identification often leads to a lack of support surrounding the experience, which in turn is associated with worse mental health outcomes (Clements & Ogle, 2009; Fondacaro et al., 1999). Addressing NSEs and post-NSE adjustment and recovery, and potentially reducing the prevalence of mental health issues, among incarcerated men may serve to improve rehabilitation and recidivism rates.

Unlike the United States of America that instituted The Prison Rape Elimiantion Act (PREA) in 2003 requiring federal, state and local correctional facilities maintain and enforce a zero-tolerance policy toward sexual assault perpetrated by staff or incarcarated individuals on other incarcerated individuals and sexual misconduct (PREA, 2003), Canada has no such legislative measure. There is limited infrastructure in Canada to address the systematic NSEs in prisons as well as histories of NSE among incarcerated individuals. The lack of support may lead incarcerated individuals to under-report NSEs for fear of retribution and revictimization, and this issue is compounded with the limited repercussions in place for perpetrators. With the responsibility to address NSEs among incarcerated men placed on the carceral system, screening measures that remove the barrier of identification and disclosure, and specifically address the behaviors of NSEs may help to recognize those who would benefit from targeted interventions and further support. Correctional educational programing on mental health within prisons has demonstrated effectiveness (Bozick et al., 2018) and the expressed willingness of incarcerated individuals to disclose and seek support highlight the importance of programing as a means through which NSEs can be addressed within prisons.

### Limitations

Despite several notable findings, the potential limitations of this study should be addressed. The use of the term “sexual boundaries” in the open-ended question could be read as ambiguous, and thus leaves room for the respondent to interpret the question in various ways influencing their response. With respect to the qualitative question, it is also important to differentiate non-responses (36%) from participants explicitly stating they have not experienced NSEs (41%). Participants who chose not to respond to the qualitative question may or may not have had NSEs and this possibility presents a very different interpretation of their response from participants who explicitly stated no NSEs. While few participants needed the questions read to them due to lack of reading skills it may also be possible that some participants chose not to respond to the qualitative question due to embarrassment around their spelling and printing skills. Childhood NSEs were measured using the label of “abuse” which may have prevented individuals who do not identify their childhood NSEs with that sexualized violence label from self-reporting experiences. A more comprehensive measure of childhood experiences could have strengthened the estimates of childhood NSEs in this sample. Notably, the assessment of childhood NSEs was added later in data collection and provided only to the third prison (i.e., Maplehurst Correctional Complex). As such, the rates are only representative of a smaller portion of our sample (*n* = 60, 30.2%) and may not be generalizable to the rest of our sample.

When interpreting all data, the influence of socially desirable response bias must be taken into consideration. For instance, some incarcerated individuals expressed that they did not want to answer some questions over fear of being “moved to the sex crimes pod/unit.” A further limitation may be the dynamics and responsiveness of the incarcerated individuals based on RA characteristics (e.g., gender, tone, appearance). Also, some participants registered for the study using a public sign-up sheet that all incarcerated individuals in their facility had access to and would thus be known by other incarcerated individuals as wanting to participate in the study. This recruitment method may have deterred some incarcerated individuals from participating. The lack of compensation offered to participants may be seen as a further limitation. Lastly, the current study did not assess mental health disparities between those with and without NSE histories. While the open ended questions indicate experiential distress for those with NSE histories, future research may want to explore disparities between these groups in this population to provide further support for programing needs.

### Future Directions

Insight into the NSE histories among incarcerated men can be utilized to improve the mental health and wellbeing of incarcerated individuals through informed policy making, prison programing, and rehabilitation efforts. Future research may be strengthened by taking into consideration known risk factors of both childhood NSEs and later criminal behavior such as social and economic inequalities. NSEs are a known risk factor in mental health issues and are an important target for rehabilitation that may serve to improve recidivism rates. Future research should be designed to explore screening measures, the development of correctional educational programing, and other counseling resources within the prison system that specifically address NSEs.
